# Development of a Xylene-Free Sample Preparation Protocol for Quantitative Proteomics of Clinically Relevant Formaldehyde-Fixed Paraffin-Embedded Needle Biopsy Samples

**DOI:** 10.3390/proteomes14020030

**Published:** 2026-06-14

**Authors:** Gontse Mabuse Moagi, Lívia Beke, Gábor Méhes, Gábor Kecskeméti, Zoltán Szabó, Lilla Turiák, Éva Csősz

**Affiliations:** 1 Proteomics Core Facility, Department of Biochemistry and Molecular Biology, Faculty of Medicine, University of Debrecen, Egyetem tér 1, H-4032 Debrecen, Hungary; mabuse.moagi@med.unideb.hu; 2 Doctoral School of Molecular Cell and Immune Biology, University of Debrecen, Egyetem tér 1, H-4032 Debrecen, Hungary; 3 Health Care Service and Units, Department of Pathology, Faculty of Medicine, University of Debrecen, Egyetem tér 1, H-4032 Debrecen, Hungary; livia.beke@med.unideb.hu (L.B.); gabor.mehes@med.unideb.hu (G.M.); 4 Department of Medical Chemistry, Albert Szent-Györgyi Medical School, University of Szeged, Dóm Square 8, H-6720 Szeged, Hungary; kecskemeti.gabor@med.u-szeged.hu (G.K.); szabo.zoltan@med.u-szeged.hu (Z.S.); 5 MTA-HUN-REN TTK Lendület (Momentum) Glycan Biomarker Research Group, HUN-REN Research Centre for Natural Sciences, Magyar Tudósok Körútja 2, H-1117 Budapest, Hungary; turiak.lilla@ttk.hu

**Keywords:** FFPE, DIA, HAIR, needle biopsy, xylene-free, mass spectrometry

## Abstract

Background: Fresh frozen tissues are considered the gold standard for proteomic analyses due to their superior preservation of protein integrity; however, their use is limited by the logistical and financial requirements of long-term cold storage. Formaldehyde-fixed paraffin-embedded (FFPE) tissues provide a practical alternative, owing to their stability and widespread availability in clinical settings. A critical step in FFPE proteomics is deparaffinization, which traditionally relies on organic solvents such as xylene, along with the efficient reversal of formaldehyde-induced crosslinks. Methods: In this study, we evaluated multiple FFPE protein extraction and digestion workflows including chaotropic, surfactant-based, and detergent-free approaches in combination with xylene-free deparaffinization strategies, using label-free data-independent acquisition (DIA) LC-MS/MS. Results: Among the tested methods, a chaotropic, reductant, and surfactant-free in-solution digestion workflow demonstrated robust protein and peptide recovery. A modified version of this protocol further improved peptide coverage while maintaining comparable protein depth. The applicability of the optimized workflow was assessed using FFPE needle biopsy samples from control, hepatic steatosis, and liver fibrosis groups. Exploratory proteomic patterns were observed across conditions, with hepatic steatosis associated with early activation of stress-response pathways, while fibrosis showed evidence suggesting altered lipid metabolism. Conclusions: Overall, this study presents a simple, xylene-free, and MS-compatible workflow for FFPE proteomics that is suitable for low-input clinical samples and may support broader application of archival tissues in proteomic research.

## 1. Introduction

Mass spectrometry (MS)-based proteomics, particularly liquid chromatography–tandem mass spectrometry (LC-MS/MS), has become a cornerstone for the comprehensive characterization of biological systems [1,2]. While global proteomic analyses of bodily fluids are widely used to investigate disease mechanisms and predict clinical outcomes, tissue-based proteomics provides essential spatial and cellular context that cannot be captured from biofluids alone [3,4].

Tissue specimens are commonly preserved as either fresh frozen or formaldehyde-fixed paraffin-embedded (FFPE) samples. Fresh frozen tissues are considered the gold standard for proteomic analyses due to their superior preservation of protein integrity; however, their use is constrained by the logistical and financial demands of long-term cold storage. By contrast, FFPE tissues represent a highly accessible and stable alternative, enabling room-temperature storage while preserving tissue architecture for histopathological assessment [5]. Although FFPE preservation can compromise quantitative reproducibility relative to fresh frozen tissues, previous studies have demonstrated substantial overlap in protein identifications and gene ontology profiles between the two approaches [6,7,8], supporting the applicability of FFPE specimens for proteomic investigations.

Proteomic analysis of FFPE tissues can be performed using scrolls or section-based sampling approaches, including laser capture microdissection (LCM) and manual macrodissection (MMD) [9,10]. Among these, MMD offers practical advantages by enabling spatially resolved sampling guided by histopathological features while avoiding the technical complexity and labor-intensive nature of LCM [11]. Consequently, MMD has become a widely adopted strategy in routine FFPE proteomics workflows.

A critical step in FFPE sample preparation is deparaffinization, which has traditionally relied on organic solvents such as xylene, followed by graded ethanol washes for rehydration [12,13]. Although effective, these methods involve hazardous reagents and multiple processing steps. Additionally, xylene exposure is associated with adverse health effects, including dizziness, headaches, nausea, and irritation of the eyes and respiratory tract [14]. Alternative strategies have, therefore, been introduced, including commercial reagents like SafeClear [15] and proprietary systems such as adaptive focused acoustics (AFA) [16]. Solvent-free or “green” deparaffinization approaches such as hot water-based methods [17,18], projected hot air deparaffinization (PHAD) [19], and oven heating [20] are prospective approaches due to their simplicity and reduced chemical usage.

Following deparaffinization, FFPE proteomic analysis requires the reversal of formaldehyde-induced crosslinks to facilitate efficient protein extraction and enzymatic digestion. These crosslinks, primarily methylene bridges, reduce protease accessibility, particularly for membrane-associated and low-abundance proteins, thereby limiting proteome coverage [21]. Crosslink reversal can be achieved through a chemical de-crosslinker such as hydroxylamine under mild conditions ([22]) or, more commonly, through the application of thermal energy (typically 95–120 °C) in combination with optimized buffer systems, a process known as heat-induced antigen/epitope retrieval (HIAR/HIER), originally developed for immunohistochemistry [21,23]. Optimized buffers often include strong detergents such as sodium dodecyl sulfate (SDS) to enhance protein solubilization. However, such detergents are incompatible with LC-MS/MS and require additional cleanup steps, including protein precipitation or filter-based buffer exchange, which can be problematic for low-input samples and may result in sample loss [10,24].

In this study, we systematically evaluated multiple FFPE protein extraction and digestion workflows in combination with solvent-free deparaffinization strategies. By comparing chaotropic, surfactant-based, and detergent-free approaches, including a modified protocol optimized for low-input material, we aim to identify robust and MS-compatible methods that maximize proteome coverage. The optimized workflow was further applied to liver needle biopsy samples across progressive stages of liver damage, demonstrating its suitability for low-input proteomics and its potential to uncover biologically relevant proteomic alterations. This work provides a practical framework for improving FFPE-based proteomics, with an emphasis on relative simplicity and compatibility with limited sample material.

## 2. Materials and Methods

### 2.1. Sample Information

FFPE sample material used for protocol optimization consisted of freely available archived patient tissue obtained from routine diagnostic procedures. The disease cohort consisted of three groups (*n* = 3 per group). Control samples were derived from histologically normal liver tissue obtained from patients investigated for suspected hepatocellular carcinoma, soft tissue lesions, or hemochromatosis, with no evidence of pathological liver changes. The steatosis group comprised cases of moderate hepatic steatosis, whereas the fibrosis group included cases with mild-to-moderate fibrosis (F1/4–F2/4) of unknown etiology. Participants were 32–72 years of age, with an overall balanced sex distribution (1:1 female-to-male ratio).

Needle biopsy specimens were fixed in 4% paraformaldehyde at 4 °C for 6 h, followed by sequential dehydration in 75%, 80%, 90%, 95%, and 100% ethanol. The resulting FFPE blocks were sectioned at a thickness of 10 μm using a HM34E rotation microtome (Epredia Hungary, Budapest, Hungary) according to standard protocols and mounted onto Superfrost™ glass slides (Epredia Hungary, Budapest, Hungary). This work was approved by the Scientific and Research Ethics Committee of the Scientific Council of Health of the Hungarian Government under the registration number of IV/8465-3/2021/EKU, date of approval: 5 November 2021.

### 2.2. Protein Extraction from FFPE Tissue Sections

All reagents used in the procedure were purchased from Sigma (Buchs, Switzerland), unless otherwise specified. Serial formaldehyde-fixed paraffin-embedded (FFPE) tissue sections (10 μm thickness) were baked at 60 °C for 2 h to prevent tissue detachment. Deparaffinization was subsequently performed using either projected hot air deparaffinization (PHAD) or hot distilled water. For PHAD, a conventional hair dryer was used at maximum power for 20 min to melt paraffin. Slides were immediately immersed in distilled water for 5 min to facilitate rehydration and solidification of residual paraffin. For the hot water method, 1 mL of distilled water at 80 °C was applied dropwise to melt and remove paraffin. A ruler was used to delineate the tissue area for consistent sampling, and the tissue samples were collected in five technical replicates with the help of a sterile scalpel. Tissue scraping was limited to excluding surrounding paraffin rather than enriching for specific histological regions.

The resulting deparaffinized tissue slices were subjected to further processing and digestion according to the following protocols.

#### 2.2.1. Approach 1: Chaotropic-Based In-Solution Digestion (App_1)

Heat-induced antigen retrieval (HIAR) was performed prior to tissue collection by immersing slides in citrate buffer (95 mM trisodium citrate, 21 mM citric acid, pH 6.0) at 75 °C for 60 min. Tissue was then scraped off, resuspended in 75 μL of 8 M urea, and solubilized by 15 cycles of sonication (1 min on, 30 s off) in a chilled (~1.5 °C) water bath. Samples were reduced with 10 mM dithiothreitol (DTT) for 30 min at 37 °C and alkylated with 15 mM iodoacetamide (IAA) for 20 min at room temperature in the dark. The solution was diluted to 4 M urea with 50 mM Tris-HCl (pH 8.0) and incubated with 40 ng Pierce Trypsin/Lys-C (Thermo Fisher Scientific, San Jose, CA, USA) for 4 h at 37 °C. Urea concentration was further reduced to 0.8 M prior to overnight digestion with 60 ng trypsin at 37 °C. Digestion was terminated by acidification with 6% trifluoroacetic acid (TFA) to pH < 3.

#### 2.2.2. Approach 2: Chaotropic, Reductant, and Surfactant-Free In-Solution Digestion (App_2)

This approach was adapted from a previously described method [25]. Collected tissue was washed with 50 μL of 100% acetonitrile (ACN) and dried using a SpeedVac (Thermo Fisher Scientific, San Jose, CA, USA). The dried sample was resuspended in 40 μL of 60 mM triethylammonium bicarbonate (TEAB) and incubated at 95 °C for 60 min. Subsequently, 10 μL of 60% ACN in 60 mM TEAB was added, followed by incubation at 75 °C for an additional 60 min. Pre-digestion was performed with 40 ng Trypsin/Lys-C for 4 h at 37 °C, followed by overnight digestion with 60 ng trypsin (16 h, 37 °C). The reaction was terminated by acidification with 6% TFA to pH < 3.

#### 2.2.3. Approach 3: On-Surface Surfactant-Based In-Solution Digestion (App_3)

This protocol was adapted with minor modifications [10]. Tissue was solubilized in 100 μL of freshly prepared buffer (80 mM HEPES, pH 8.0, 80 mM DTT, 4% SDS). Samples underwent two rounds of sonication (each round consisted of 15 cycles: 1 min on, 30 s off) in a chilled (~1.5 °C) water bath, followed by incubation at 99 °C for 1 h. After centrifugation (15 min, 13,000× *g*, RT), the supernatant was collected and alkylated with IAA to a final concentration of 15 mM for 30 min in the dark. Proteins were precipitated with four volumes of ice-cold acetone and incubated overnight at −20 °C. Samples were centrifuged (40 min, 16,000× *g*, 4 °C), and pellets were washed twice with 80% ice-cold acetone. Pellets were resuspended in 25 μL digestion buffer (1.5 M urea, 100 mM HEPES, pH 8.0) using sonication (3 cycles). Trypsin/Lys-C digestion (1:30, *w*/*w*) was performed for 4 h at 37 °C (600 rpm), followed by dilution to 0.75 M urea and overnight trypsin digestion (1:20, *w*/*w*). Digestion was quenched with TFA (pH < 3).

#### 2.2.4. Approach 4: On-Surface Surfactant-Based Digestion (App_4)

This method was adapted with minor modifications [26]. HIAR was performed as described in App_1. Reduction was carried out by applying a 3 μL droplet (20% glycerol, 0.1% SDS, 5 mM DTT) and incubating for 40 min at 37 °C. Alkylation was performed using a 3 μL droplet containing 10 mM IAA in 25 mM ammonium bicarbonate (ABC) for 20 min at room temperature in the dark. Pre-digestion was conducted in two cycles using 3 μL droplets (20% glycerol, 40 ng Trypsin/Lys-C in 50 mM ABC) for 40 min at 37 °C in a humidified chamber. Final digestion was performed in three cycles with 200 ng trypsin under the same conditions. Peptides were extracted by repeated pipetting (5×) with 3 μL of 10% acetic acid and collected into Eppendorf LoBind™ tubes (Eppendorf SE, Hamburg, Germany).

#### 2.2.5. Approach 2M: Chaotropic and Surfactant-Free Buffer with Reduction/Alkylation (App_2M)

This modified protocol incorporated reduction and alkylation into App_2. Following incubation at 95 °C in 60 mM TEAB, samples were reduced using 10 μL of 60% ACN containing 80 mM DTT and incubated at 75 °C for 60 min. Alkylation was performed with 15 mM IAA for 30 min at room temperature in the dark. Pre-digestion (40 ng Trypsin/Lys-C, 4 h at 37 °C) and subsequent steps were identical to those described for App_2.

Following all protocols, peptides were purified using C18 PierceTips (Thermo Fisher Scientific, San Jose, CA, USA), dried, and stored at −20 °C until further analysis.

### 2.3. Equal Loading of Samples

Peptide concentrations were estimated by measuring absorbance at 280 nm (A280) using a NanoDrop ND-1000 spectrophotometer (Thermo Fisher Scientific, San Jose, CA, USA), with the conversion factor set to “1 Abs = 1 mg/mL”. Samples were normalized to a final concentration of 80 ng/μL in 1% formic acid (FA) prior to analysis.

### 2.4. Chromatographic and Mass Spectrometric Condition

Digested samples were redissolved in 10 µL 1% FA and analyzed as single-shot DIA analyses. For method optimization, the LC-MS analysis was carried out on an EASY nLC-1200 coupled to Orbitrap Fusion (both from Thermo Fisher Scientific, San Jose, CA, USA). First, 5 µL of sample was injected at a constant flow of 1 µL/min solvent A to a 5 µm, 180 µm × 20 mm NANOACUITY™ trap column packed with ACUITY UPLC™ Symmetry C18 particles (Waters, Milford, MA, USA). Then, analytical nanoflow separations were achieved using a 1.7 µm, 75 µm × 150 mm ACUITY UPLC™ M-Class Peptide BEH C18 column (Waters, Milford, MA, USA). Peptides were eluted at a flow rate of 300 nL/min at a gradient of 0–2% solvent B over 2 min, followed by a rise to 25% of solvent B over 52 min and then to 38% solvent B over 11 min. Thereafter, solvent B was increased to 90% over 4 min and held for 3 min, after which the system returned to 2% solvent B in 1 min for a 2 min hold on. For mass spectrometry analyses, the nanoelectrospray ion source (NSI) (Nanospray Flex™, Thermo Fisher Scientific, San Jose, CA, USA) was operated at 2.3 kV in the positive-ion mode, with an ion-transfer tube set to 275 °C. Following MS1 spectra collection at 120,000 resolution in the range of 350–1200 *m*/*z*, precursor ions were fragmented at fixed isolation windows of 12 *m*/*z* with 55 scan events, in DIA mode, with HCD fragmentation using a 30% normalized collision energy to produce corresponding MS2 spectra. Product ions were detected in the Orbitrap analyzer with an AGC target of 2.0 × 10^5^ and a maximum injection time of 60 ms, 30,000 resolutions in the range of 145–1450 *m*/*z*.

Case study LC-MS analysis was carried out on a Waters ACQUITY UPLC M-Class LC system (Waters, Milford, MA, USA) coupled to Orbitrap Exploris™ 240 Mass Spectrometer (Thermo Fisher Scientific, San Jose, CA, USA). First, 2 µL of sample was injected at a constant flow of 7 µL/min solvent A to a Symmetry^®^ C18 (100 Å, 5 µm, 180 µm × 20 mm) trap column (Waters, Milford, MA, USA). Then, analytical nanoflow separations were achieved using an ACQUITY UPLC^®^ M-Class Peptide BEH C18 analytical column (Waters, Milford, MA, USA) (130 Å, 1.7 µm, 75 µm × 250 mm). Peptides were eluted at a flow rate of 200 nL/min at a gradient of 3–8% solvent B over 1 min, followed by a rise to 32% of solvent B over 81 min and then to 40% solvent B over 3 min. Thereafter, solvent B was increased to 90% over 1 min and held for 2 min, after which the system returned to 3% solvent B in 1 min for a 15 min hold on. For mass spectrometry analyses, the nanoelectrospray ion source (NSI) (Nanospray Flex™, Thermo Fisher Scientific, San Jose, CA, USA) was operated at 1.8 kV in the positive-ion mode, with an ion-transfer tube set to 280 °C. Following MS1 spectra collection at 30,000 resolution in the range of 380–985 *m*/*z*, precursor ions were fragmented at fixed isolation windows of 10 *m*/*z* with 60 scan events, in DIA mode, with HCD fragmentation using a 28% normalized collision energy to produce corresponding MS2 spectra. Product ions were detected in the Orbitrap analyzer with an AGC target of 2.0 × 10^6^ and a maximum injection time of 40 ms, 11,250 resolutions, and the DIA *m*/*z* range was set to auto.

### 2.5. Data Analysis

Raw files were processed using DIA-NN (version 2.2) [27,28] in library-free search mode (Table S1). This tool supports endogenous peptides-based retention time alignment; as such, no indexed retention time (iRT) peptides were added in the samples. A human protein sequence FASTA database (20420 reviewed entries) was downloaded from UniProtKB on 26 July 2025, and ‘FASTA digest’ and ‘Deep-learning spectra’ were enabled for in silico library generation. Other parameters included: Trypsin/P with maximum 2 missed cleavage; 3 maximum number of variable modifications; protein N-terminal M excision on; and C-Carbamidomethyl was set as fixed modification for all approaches but App_2, while M oxidation, and N-terminal acetylation were set as variable modification. The XICs output was enable and all other parameters were kept as default.

The DIA-NN PARQUET output files were imported into the DEF workflow [29] together with the corresponding XICs report and report.pg_matrix files. Quantification parameters were maintained at default settings, and the HaoGroup contaminant library [30] was applied. Protein quantities were defined using the DIA-NN PG.MaxLFQ algorithms.

To improve quantification reliability, proteins were required to be supported by at least two peptides; thus, single-hit identifications were excluded. Peptides were retained only if they contained a minimum of four consecutive b- or y-ions. Furthermore, proteins were required to be identified in at least two out of three biological replicates to minimize false-negative identifications.

Statistical comparisons for protocol optimization were performed using an unpaired t-test with Welch’s correction in GraphPad Prism (version 8.0.1 for Windows, GraphPad Software, San Diego, CA, USA, www.graphpad.com, accessed on 11 April 2026). Results were considered significant at *p* < 0.05.

Principal component analysis (PCA) and protein ID overlap (%) calculations were performed in Python (version 3.12.7) using the sklearn.decomposition and pandas libraries, respectively. Venn diagrams were generated using the IntersectMe web application (version 9.7.0).

For the diseased liver case study analysis, we applied our optimized digestion method for the examination of liver needle biopsies, where the group-level contrasts were defined within the DEF “Group-Visuals” module [29] to enable direct comparison between experimental conditions. Three comparisons (control, liver fibrosis, and hepatic steatosis) were specified, allowing a maximum of one missing value per replicate. The resulting datasets were exported for analysis in MetaboAnalyst 5 [31]. Given the low-input nature of FFPE needle biopsy samples, missing values were expected, particularly for low-abundance proteins. Data were filtered using a low-variance filter, and missing values were imputed using a left-censored approach by replacing them with one-fifth of the minimum positive intensity value. Subsequently, data were median-normalized and log10-transformed. Normalized protein abundances were used for statistical analysis. Differential abundance analysis was carried out on normalized and transformed data using the limma model implemented in MetaboAnalyst, with multiple testing correction applied using the Benjamini–Krieger false discovery rate (FDR) method. Proteins were classified as differentially abundant proteins (DAPs) if they exhibited a q-value < 0.05 and an absolute log_2_ fold change greater > 1.5.

DAPs were exported from MetaboAnalyst, and tertiary analysis was performed using the Ingenuity Pathway Analysis (IPA) platform (QIAGEN Inc., Redwood City, CA, USA) to obtain functional annotations, protein interaction networks, canonical pathways, and associated biological processes. Pathway enrichment significance was evaluated using right-tailed Fisher’s exact tests, whereas activation or inhibition states were predicted using IPA-generated z-scores under default parameters. Upstream regulators, downstream biological processes, and their predicted causal interactions were identified using the Ingenuity Knowledge Base, with z-scores calculated according to the directional abundance changes of DAPs.

## 3. Results and Discussion

Optimization experiments were conducted in three consecutive steps. First, the sample preparation efficiency was evaluated from a limited tissue area (~0.025 mm^3^) using four FFPE processing approaches (App_1–4). This was followed by the application of the best two approaches (App_2 and App_3) to assess the qualitative and quantitative differences between the results obtained by the application of the two selected approaches. Based on the results, App_2 was further optimized, and in the final step, the modified form of App_2, the App_2M, was used in a case study to examine biological differences characteristic of different forms of liver disease.

### 3.1. Xylene-Free Methods of FFPE Sample Preparation for Proteomics Analyses

Our goal was to develop a simpler, safer, and economically feasible protocol without the need for the application of often-expensive instrumentation. While deparaffinization by water has been employed in proteomics studies [17,18], PHAD is yet to gain proteomics utility. Using two types of xylene-free deparaffinization, various protocols were evaluated for deep coverage of the FFPE tissue proteome in an effort to maximize the number of protein groups (PGs) obtained given the limited tissue sample availability (approximately 0.025 mm^3^) in the case of needle biopsies. Approaches 1–4 include a wide range of widely applied non-commercial techniques for reversing the crosslinked nature of FFPE tissues, including heat-induced antigen retrieval (HAIR) in citrate, TEAB, and SDS-containing buffers. Based on the number of peptides and PGs (Figure S1) identified, App_2 (TEAB) and App_3 (SDS-based) resulted in the highest numbers and were chosen for further experiments.

#### Method Refinement

**Figure 1:**
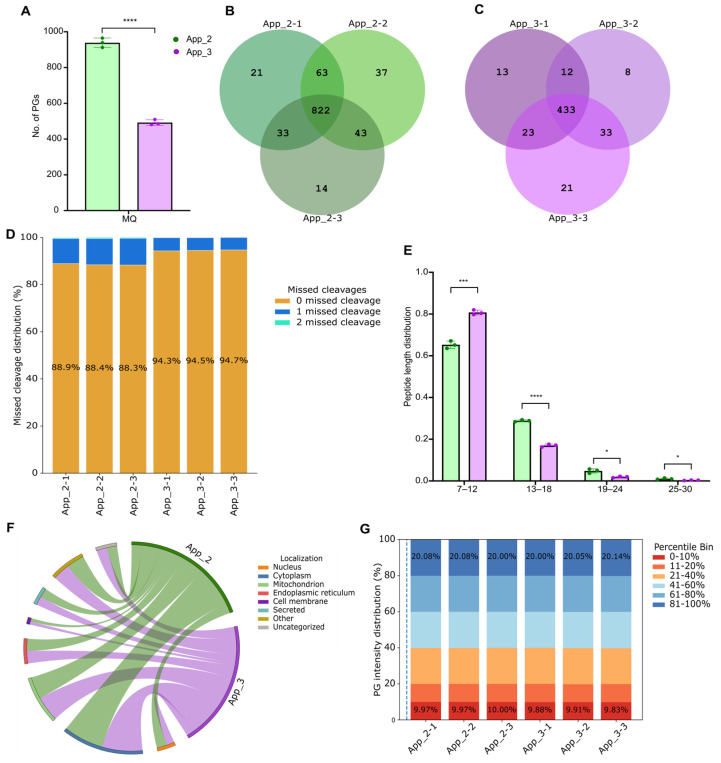
Comparative evaluation of Approach 2 and Approach 3 for FFPE proteomic analysis in three technical replicates. (**A**) Comparison of the number of protein groups (PGs) identified using Approach 2 (App_2) and Approach 3 (App_3). Statistical significance was assessed using an unpaired t-test with Welch’s correction (**** *p* < 0.0001; *** *p* < 0.001; * *p* < 0.05). (**B**,**C**) Venn diagrams of PG overlap across three technical replicates for (**B**) App_2 and (**C**) App_3. (**D**) Distribution of identified peptides according to the number of missed cleavages (0–2), reflecting digestion efficiency across both approaches. (**E**) Peptide length distribution presented as the number of identified peptides within defined amino acid length bins (7–12, 13–18, 19–24, and 25–30 residues). (**F**) The distribution of identified proteins across subcellular compartments for App_2 and App_3. (**G**) Distribution of PG intensities across percentile bins (0–10, 11–20, 21–40, 41–60, 61–80, and 81–100), highlighting detection sensitivity between the two approaches. “−1, −2, or −3” in the names of App_2 and App_3 refer to the technical replicates.

**Figure 2:**
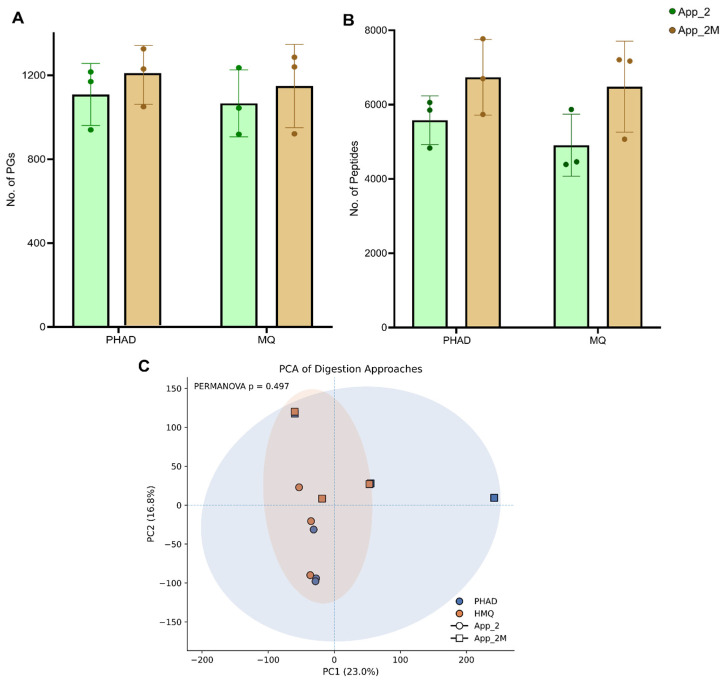
Comparative analysis of App_2 and App_2M using different deparaffinization methods in three technical replicates. App_2 and App_2M were evaluated following deparaffinization with projected hot air deparaffinization (PHAD) or hot Milli-Q water (HMQ). (**A**) Mean number of identified peptides and (**B**) protein groups (PGs), presented with standard deviation. (**C**) Principal component analysis of protein intensities derived from three replicates per approach and deparaffinization condition, ellipses highlight the spread of PHAD (blue) and HMQ (orange) clusters.

Three randomly selected regions from two consecutive needle biopsy slides were digested with App_2 and App_3, providing three technical replicates in each case. Proteomics data were processed in DEF, and the peptide and protein lists were evaluated (Table S2). The number of PGs from App_2 and App_3 differed significantly (App_2, 939 PGs; App_3, 492 PGs) (Figure 1A). A substantial proportion of proteins was consistently detected across all replicates, while only a subset was identified in individual samples (Figure 1B, Figure 2C and Figure S2A), which highlights the technical robustness of App_2 and App_3. Although the quantitative profiles obtained with App_2 and App_3 were moderately correlated (Pearson r = 0.69) (Figure 2B), substantial abundance differences were observed between workflows. Across proteins quantified by both methods, App_2 yielded approximately 1.5-fold higher abundances on average, and over half of the shared proteins differed by more than twofold (Figure 1C). These findings suggest that the workflows produce broadly similar proteome profiles but differ considerably in protein extraction and/or recovery efficiency.

Studies have shown that using dual protease with trypsin and Lys-C has a positive effect on proteome coverage [32]; as such, a trypsin/Lys-C mix was used for all digestion methods in order to maximize coverage and ensure a better comparability. While in more than 88% of the peptides, 0 missed cleavages were observed in both approaches, App_3 showed an approximately 6% times higher 0 missed cleavage rate (Figure 1D). This increased cleavage efficiency could be due to extensive reduction and homogenization with sonication in HEPES buffer containing DTT and SDS. This was also evident at the physicochemical level, with App_3 showing an increased proportion of shorter peptides (7–12 amino acids) (Figure 1E), which suggests enhanced proteolytic cleavage efficiency. Nonetheless, the distribution of proteins among subcellular locations was nearly identical (Figure 1F), suggesting that the two approaches were not biased toward any specific cellular compartment (Figure S2D).

The distribution of quantified proteins across intensity percentiles revealed highly similar quantitative dynamic ranges (Figure 1G). Both approaches demonstrated a comparable proportion of proteins in lower- and higher-percentile bins. This indicates that App2 and App_3 provide similar sensitivity and proteome depth.

Considering the number of post-translationally modified peptides, no significant difference could be observed between App_2 and App_3 (Figure S3), which indicates their similar performance in proteoform identification.

As App_2 provides more PGs with fewer steps and is easier to perform, we chose App_2 for further experiments. Based on the better cleavage efficiency observed in App_3, in the next step, we modified App_2 by including reduction and alkylation as in App_3. The number of identified PGs showed a modest increase with the App_2M protocol compared to App_2 (Table S3). The mean PG counts were 1108 and 1202 for PHAD and 1066 and 1149 for HMQ-based deparaffinization, respectively. However, these differences did not reach statistical significance (Figure 2A). A similar pattern was seen with the number of identified peptides (Figure 2B). The principal component analysis of protein intensities from App_2 and App_2M showed no distinct clusters (Figure 2C); additionally, there was no significance in either the use of a hairdryer or deparaffinization with hot water.

We further evaluated a peptide-to-protein distribution to highlight approach depth (Table 1).

Although the dependence of PG identification on sequence counts was comparable across methods (Figure S4), App_2M showed an enrichment of protein groups supported by multiple unique peptides, particularly for proteins identified by 6–20 unique peptides. This change was significant, particularly for HMQ-based deparaffinization, indicating improved peptide coverage per protein group (Figure S4). Our results show that modifying App_2 produced a protocol that delivers strong performance. While App_2M incorporates additional reduction and alkylation steps compared to App_2, it remains less labor-intensive than App_3 and yields superior peptide and protein group identification.

Extraction buffers containing chaotropes or detergents are known to interfere with the protease activity of trypsin [33], which results in incomplete protein digestion and, consequently, in lower proteome coverage due to oversampling of different cleavage forms of abundant peptides. However, with the inclusion of cleanup steps, surfactant-containing extraction buffers enhance trypsin activity and increase digestion efficiency [32,34,35]. This could be seen in the analysis of App_3’s missed cleavage frequencies. The resultant low number of peptides could be due to loss during acetone precipitation. This suggests that App_3 could benefit from further refinements. However, considering time and simplicity, the improvement of App_2 was in line with our objective of a simple and user-friendly protocol.

Our results indicate that both App_2 and App_2M have good performance with respect to the proteome integrity of the extracted proteins. They also show consistent protein abundances between technical replicates and a low percentage of missed cleaved peptides, possibly due to the use of a four-hour Lys-C pre-digestion prior to overnight digestion with trypsin [36]. There is, however, a slight improvement in terms of the number of quantified peptides and proteins and sequence coverage.

With our modified approach, we could identify about 1200 proteins, which is consistent with previous reports of low-input FFPE proteomics using data-independent acquisition. Studies employing biopsy-scale or laser-microdissected FFPE samples typically report proteome depths in the range of ~800–2000 proteins [16,18,37], reflecting the strong influence of sample input on proteome coverage.

### 3.2. Case Study—Examination of Liver Needle Biopsy Samples

Three biological replicates of FFPE needle biopsy samples (~0.03–0.05 mm^3^) were analyzed with App_2M for each liver condition (fibrosis and hepatic steatosis), together with matched controls. The total number of identified proteins and peptides is summarized in Table 2, while detailed lists of identified peptides and proteins are provided in Table S4 and Table S5, respectively. Differences in the number of identified protein groups between control and disease samples may reflect both the biological heterogeneity and variable protein recovery inherent to low-input FFPE proteomics workflows.

Prior to downstream analysis, three samples (one from each condition) were excluded due to poor data quality. These samples exhibited a markedly reduced number of identified proteins and peptides, along with a disproportionately high contribution of hemoglobin and keratin (>50% of total signal), indicative of contamination (Figure S5). Consistently, these samples appear as outliers, primarily for the hepatic steatosis and fibrosis groups, in principal component analysis (PCA) and heat maps (Figure S6).

**Figure 3:**
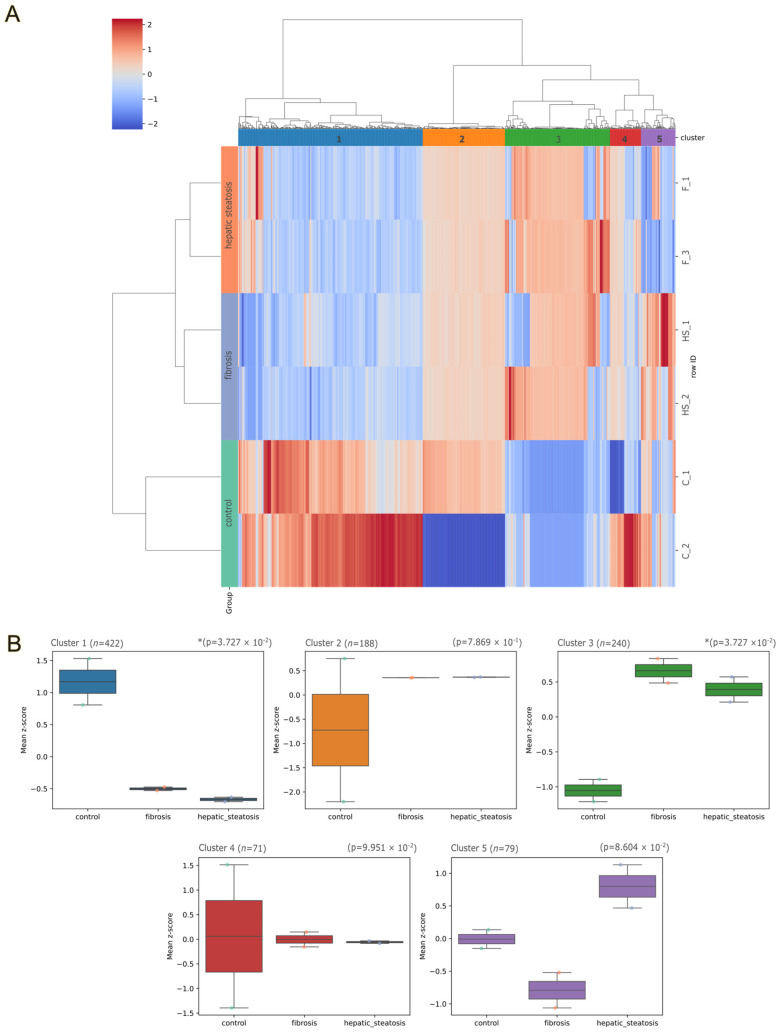
Protein abundance patterns across control (*n* = 2), hepatic steatosis (*n* = 2), and liver fibrosis (*n* = 2) groups. (**A**) Hierarchically clustered heat map of protein abundances across the three conditions. Proteins are grouped into five clusters (Clusters 1–5) based on similarity in abundance profiles. Values represent Z-score-normalized intensities. (**B**) Box plots showing the distribution of mean Z-scores for each protein cluster (Clusters 1–5) across the three conditions (* *p* < 0.05), the color of box plot correspond to their cluster, while scatter points correspond to their respective condition group.

Relative protein abundance patterns were visualized using a clustered heat map, revealing clear condition-dependent segregation (Figure 3A). Proteins with similar abundance profiles were grouped into five distinct clusters (Figure 3B).

**Figure 4:**
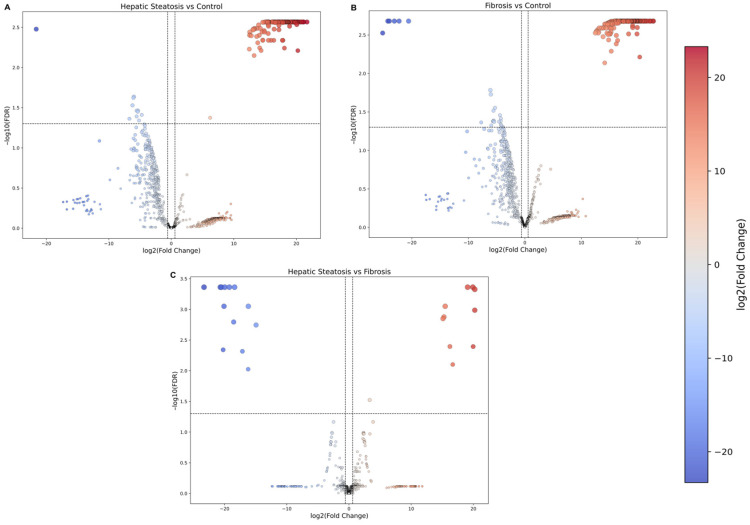
Differentially abundant proteins identified by limma analysis. (**A**–**C**) Volcano plots showing differentially abundant proteins between (**A**) hepatic steatosis and control (*n* = 2), (**B**) liver fibrosis and control (*n* = 2), and (**C**) hepatic steatosis and liver fibrosis (*n* = 2). Differential abundance was defined using a log_2_ fold-change (log_2_FC) threshold of ≥1.5 (horizontal dashed lines) and a Benjamini–Hochberg false discovery rate (FDR) q-value < 0.05 5 (vertical dashed lines). Proteins with increased abundance are indicated in red, whereas proteins with decreased abundance are shown in blue.

Differential abundance analysis using the limma model identified proteins with significant changes between conditions. Applying a log_2_ fold-change (log_2_FC) threshold of ≥1.5 and a Benjamini–Hochberg false discovery rate (FDR) q-value < 0.05, multiple proteins were found to be statistically differentially abundant across the disease states (Table S6). In comparison to the controls, the hepatic steatosis group exhibited 11 proteins with decreased abundance and 147 proteins with increased abundance (Figure 4A). In the fibrosis group relative to the controls, 23 proteins were found to be increased, while 151 were decreased in abundance (Figure 4B). When comparing hepatic steatosis to fibrosis, 11 proteins showed reduced abundances and 13 increased abundances (Figure 4C).

**Figure 5:**
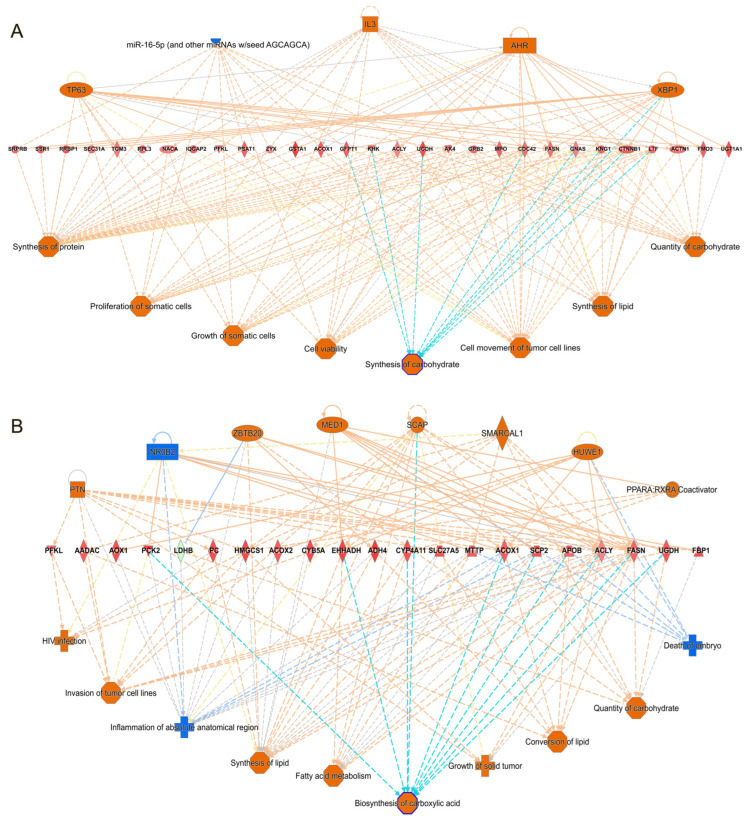
IPA regulator-effector networks of differentially abundant proteins. Regulator-effector networks generated by Ingenuity Pathway Analysis (IPA) for hepatic steatosis versus control (**A**) and fibrosis versus control (**B**). Node color reflects predicted activity state, with red indicating activation and blue indicating inhibition. Edges represent causal relationships, where orange lines denote predicted activation and blue lines denote predicted inhibition of downstream targets. Yellow edges indicate relationships that are inconsistent with the observed abundance patterns in the dataset.

The list of differentially abundant proteins (DAPs) was further interrogated using regulator effector network analysis in Ingenuity Pathway Analysis (IPA). Networks were generated separately for hepatic steatosis versus control (Figure 5A) and fibrosis versus control (Figure 5B) using the IPA Core Analysis with default settings. Predicted upstream regulators, downstream biological functions, and their causal relationships were inferred based on the Ingenuity Knowledge Base, with activation states assigned using z-scores derived from the directionality of protein abundance changes. Detailed lists of all molecules and their respective relationships are provided in Table S7.

Consistent with the comparison of fibrosis and control, downstream functional annotations predicted increased lipid synthesis, carbohydrate metabolism, and cellular growth-related processes, including the proliferation and viability of somatic cells, indicating that metabolic reprogramming is already established at the steatosis stage. However, the upstream regulatory landscape differed between hepatic steatosis and control, with predicted activation of TP63, IL3, AHR, and XBP1 alongside the inhibition of miR-16-5p and related microRNAs. These regulators are primarily associated with cellular stress responses, inflammatory signaling, and the transcriptional control of protein synthesis [38,39,40], which suggests a broader regulatory input compared to the more lipid-centric SCAP-driven network observed in the fibrosis vs. control regulator-effector networks.

At the protein level, multiple enzymes involved in the synthesis and quality of carbohydrates [41], lipid biosynthesis [42,43], and protein production [44] were interconnected, which supports a coordinated upregulation of biosynthetic pathways. Notably, functions such as synthesis of protein and synthesis of carbohydrate were prominently activated in hepatic steatosis vs. control, in contrast to fibrosis vs. control, where fatty acid metabolism and lipid-specific pathways were more dominant. Additionally, predicted activation of cell movement and tumor-associated processes indicates early engagement of pathways linked to cellular remodeling and adaptation.

Comparatively, while both networks characteristic of hepatic steatosis and fibrosis share activation of lipid metabolic processes, the latter network appears more specialized toward fatty acid metabolism and lipogenesis under the control of SCAP-/SREBP-related mechanisms [45]. By contrast, the hepatic steatosis-control network reflects a broader, upstream-driven response integrating inflammatory cues (IL3), environmental sensing (AHR), endoplasmic reticulum stress (XBP1), and microRNA-mediated regulation [38,46,47]. This suggests that hepatic steatosis represents an earlier, more heterogeneous metabolic and stress-adaptive state that precedes the more focused and transcriptionally coordinated lipid metabolic reprogramming observed in fibrosis.

Overall, these findings support a model in which hepatic steatosis initiates widespread metabolic and regulatory alterations, including activation of protein and carbohydrate biosynthesis, which subsequently converge into a more defined lipid-centric metabolic phenotype during progression to fibrosis.

Canonical pathway analysis (Figure S7) further supported the regulator-effector network findings, demonstrating consistent enrichment of detoxification and metabolic pathways in both hepatic steatosis and fibrosis compared with control samples, with differences primarily in activation magnitude and pathway emphasis. Phase I (functionalization) and Phase II (conjugation) xenobiotic metabolism pathways were among the most strongly activated in both conditions, which indicates enhanced hepatic detoxification and metabolic adaptation. This is in agreement with the predicted activation of stress- and environment-responsive regulators, such as AHR, observed in the network characteristic to hepatic steatosis (Figure 5A), supporting a role for xenobiotic and oxidative stress signaling in disease progression.

Consistent with the lipid metabolic reprogramming observed in the regulator-effector networks (Figure 5A,B), pathways related to peroxisomal function, including peroxisomal protein import and lipid metabolism, were significantly enriched, reflecting increased fatty acid handling and β-oxidation demands [48]. Similarly, enrichment of bile acid and bile salt metabolism further supports alterations in lipid and cholesterol homeostasis [49] across both conditions. The activation of retinoic acid signaling aligns with the upstream regulatory patterns identified in Figure 5A, suggesting involvement of nuclear receptor-mediated transcriptional programs linking metabolic adaptation with inflammatory and differentiation processes.

In addition, the enrichment of neutrophil degranulation corroborates the inflammatory component identified in the network analysis, indicating engagement of innate immune responses [50]. Notably, fibrosis exhibited generally higher activation scores across several pathways, which supports a more pronounced and functionally focused metabolic and immune response.

Overall, these pathway-level alterations reinforce the network-based observations, indicating that hepatic steatosis is characterized by early activation of stress-responsive, detoxification, and biosynthetic pathways under a broad upstream regulatory landscape, whereas fibrosis reflects a more pronounced and transcriptionally coordinated metabolic reprogramming, particularly centered on lipid metabolism.

A direct comparison between fibrosis and hepatic steatosis using IPA canonical pathway analysis was not feasible due to the absence of robust pathway predictions. Several pathways lacked sufficient information for activity state inference, while others did not meet the inclusion criteria, as fewer than four analysis-ready molecules were mapped to the respective pathways. This limitation is likely attributable to the relatively small number of differentially abundant proteins identified in this comparison (13 decreased and 11 increased in abundance).

The present findings are broadly consistent with emerging proteomic evidence indicating that hepatic steatosis is characterized by widespread metabolic and regulatory reprogramming, rather than a purely lipid-centric phenotype [51,52]. Several studies have demonstrated early activation of central carbon metabolism, protein synthesis, and endoplasmic reticulum stress pathways, including XBP1-mediated unfolded protein response signaling, supporting the notion of a stress-adaptive and biosynthetically active state in steatosis.

In agreement with this, inflammatory and cytokine-associated regulatory inputs [53], as well as microRNA-mediated derepression of proliferative pathways [54], have been reported in early disease stages, reinforcing the concept of a heterogeneous upstream regulatory landscape.

By contrast, progression to fibrosis has been associated with a more focused metabolic phenotype dominated by lipid metabolism and SCAP-/SREBP-driven lipogenic signaling, which suggests a transition from a broadly adaptive to a more specialized metabolic program [55].

However, some studies report earlier activation of lipogenic pathways, indicating that lipid metabolism may already be engaged during steatosis, albeit at lower magnitude [45]. Additionally, the role of regulators such as AHR appears to be context-dependent, reflecting both adaptive and pathogenic functions [56].

Collectively, these findings support a model in which hepatic steatosis represents an early, metabolically flexible state integrating stress, inflammatory, and biosynthetic responses, which progressively converges toward a lipid-centered metabolic network during fibrosis.

## 4. Conclusions

Formaldehyde-fixed paraffin-embedded (FFPE) tissues represent one of the most valuable resources for clinical and translational research. However, their broader application in proteomics remains limited by the lack of standardized, robust, and clinically accessible sample preparation workflows. In this study, four extraction and digestion workflows for FFPE tissue proteomics, all employing xylene-free deparaffinization, were systematically compared with respect to proteome depth and data quality.

Our findings demonstrate that a chaotropic-, reductant-, and surfactant-free in-solution digestion workflow (App_2) provides a high peptide and protein yield, together with reliable tryptic digestion efficiency. A modified version of this workflow (App_2M) further improved peptide coverage while maintaining comparable protein depth and overall data quality. Importantly, although traditional chemical modifications are required, particularly reduction and alkylation with DTT and IAA, respectively, the protocol does not require specialized equipment, which makes it well suited for implementation in routine laboratory settings.

Evaluation of post-translational modifications (PTMs), particularly acetylation and methionine oxidation, showed no apparent differences between App_2 and App_3 under the present experimental conditions. However, PTM analyses were performed on non-enriched samples, which limits the depth and sensitivity of PTM characterization. Accordingly, further studies incorporating dedicated PTM enrichment strategies are warranted to more comprehensively assess the suitability of these workflows for modification-specific proteomic analyses.

To assess practical applicability, the optimized workflow was applied to low-input FFPE needle biopsy samples from a small cohort comprising hepatic steatosis, liver fibrosis, and control specimens. Despite the inherent challenges associated with low-input FFPE proteomics, including missing values and limited proteome depth, the workflow generated reproducible datasets and enabled the detection of biologically plausible proteomic differences between sample groups. Nevertheless, the cohort size was not designed or powered for biological discovery, and the observed disease-associated signatures should be interpreted with caution. Rather than providing definitive insights into the molecular mechanisms underlying steatosis or fibrosis, this case study serves primarily as a proof-of-concept demonstration of the workflow’s technical capability to generate informative proteomic data from limited FFPE biopsy material.

Taken together, these findings highlight a simple, xylene-free, and mass spectrometry-compatible workflow that supports efficient proteomic analysis of FFPE tissues. With further validation and optimization, particularly in the context of PTM-enriched analyses and larger clinical cohorts, this approach may provide a practical foundation for routine research applications and future clinical or diagnostic implementation.

## Figures and Tables

**Table 1:** Percentage of proteins identified by unique peptides. The columns show the percentage of proteins identified by the number of unique peptides defined in the rows in case of different methods. Differences were calculated based on the comparison between App_2M and App_2. PHAD: projected hot air deparaffinization, HMQ: deparaffinization with hot Milli-Q water.

Number of Unique Peptides per Protein	PHAD-App_2	PHAD-App_2M	Change	HMQ-App_2	HMQ-App_2M	Change
1	40.88%	37.89%	−2.99%	42.48%	37.76%	−4.72%
2	17.57%	17.25%	−0.32%	17.80%	16.92%	−0.88%
3	10.08%	10.15%	0.07%	10.39%	10.88%	0.49%
4	6.59%	7.30%	0.71%	7.41%	6.72%	−0.69%
5	5.05%	5.59%	0.54%	4.47%	4.68%	0.21%
6–20	17.06%	18.73%	1.67%	15.45%	19.62%	4.17%
>20	2.77%	3.09%	0.32%	2.00%	3.41%	1.41%

**Table 2:** Summary of the number of peptide and protein identifications.

Sample	No. of Peptides (1% FDR)	No. of PGs
LF-1	26,416	2590
LF-2	2931	452
LF-3	26,266	2583
SH-1	25,593	2583
SH-2	26,609	2589
SH-3	2470	422
C-1	11,360	1161
C-2	7708	1113
C-3	571	83

## Data Availability

Metadata regarding the experiments are presented in .tsv format as metadata/sdrf. The mass spectrometry proteomics data generated in this study have been deposited in the ProteomeXchange Consortium via the MassIVE repository under accession number PXD078226 (MassIVE accession: MSV000101787).

## Supplementary Materials

The following supporting information can be downloaded at: https://www.mdpi.com/article/10.3390/proteomes14020030/s1, Figure S1: A global comparison of App_1–App_4; Figure S2: Overlap comparison of App_2 and App_3; Figure S3: Ratio of post-translational modified peptides; Figure S4: Proportion of proteins identified by unique peptides; Figure S5: Pie chart showing intensity distribution of protein groups (PGs); Figure S6: Heat map and principal component analysis (PCA) of study samples; Figure S7: Heat map of the top 10 canonical pathways derived from differentially abundant proteins (DAPs) in hepatic steatosis and fibrosis; Table S1: DIA-NN 2.2 precursor ion generation, output, and algorithm parameters; Table S2: App_2 and App_3 DEF group-level outputs; Table S3: App_2 and App_2M DEF group-level outputs; Table S4: Liver disease case study stripped peptide sequences; Table S5: Liver disease case study DEF group-level outputs; Table S6: Differentially abundant proteins using a limma model; Table S7: Regulator-effector network molecules and relationships.

## Abbreviations

The following abbreviations are used in this manuscript:

LC-MS/MS	Liquid chromatography–tandem mass spectrometry
FFPE	Formaldehyde-fixed paraffin-embedded
LCM	Laser capture microdissection
MMD	Manual macrodissection
AFA	Adaptive focused acoustics
PHAD	Projected hot air deparaffinization
SDS	Sodium dodecyl sulfate
SDC	Sodium deoxycholate
App	Approach
HIAR	Heat-induced antigen retrieval
HIER	Heat-induced epitope retrieval
DTT	Dithiothreitol
TFA	Trifluoroacetic acid
ACN	Acetonitrile
TEAB	Triethylammonium bicarbonate
ABC	Ammonium bicarbonate
FA	Formic acid
NSI	Nanoelectrospray ion source
iRT	Indexed retention time
PCA	Principal component analysis
FDR	False discovery rate
DAPs	Differentially abundant proteins
IPA	Ingenuity Pathway Analysis
