# Supplementary material for: Development of a Xylene-Free Sample Preparation Protocol for Quantitative Proteomics of Clinically Relevant Formaldehyde-Fixed Paraffin-Embedded Needle Biopsy Samples
Source: Proteomes. 2026 Jun 14;14(2):30. doi: 10.3390/proteomes14020030 (PMC13306278; doi:10.3390/proteomes14020030)
Supplement: Supplementary file 1 [file proteomes-14-00030-s001.zip › Supplementary Figures.pdf]

Development of a Xylene-Free Sample Preparation Protocol for Quantitative Proteomics of Clinically Relevant Formaldehyde-Fixed Paraffin-Embedded Needle Biopsy Samples

Gontse Mabuse Moagi [1,2], Lívia Beke [3], Gábor Méhes [3], Gábor Kecskeméti [4], Zoltán Szabó [4], Lilla Turiák [5] and Éva Csősz [1,*]

[1] Proteomics Core Facility, Department of Biochemistry and Molecular Biology, Faculty of Medicine, University of Debrecen, Egyetem tér 1, H-4032 Debrecen, Hungary
[2] Doctoral School of Molecular Cell and Immune Biology, University of Debrecen, Egyetem tér 1, H-4032 Debrecen, Hungary
[3] Health Care Service and Units, Department of Pathology, Faculty of Medicine, University of Debrecen, Egyetem tér 1, H-4032 Debrecen, Hungary
[4] Department of Medical Chemistry, Albert Szent-Györgyi Medical School, University of Szeged, Dóm Square 8, H-6720 Szeged, Hungary
[5] MTA-HUN-REN TTK Lendület (Momentum) Glycan Biomarker Research Group, HUN-REN Research Centre for Natural Sciences, Magyar Tudósok Körútja 2, H-1117 Budapest, Hungary
* Correspondence: cseva@med.unideb.hu

Supplementary Figures

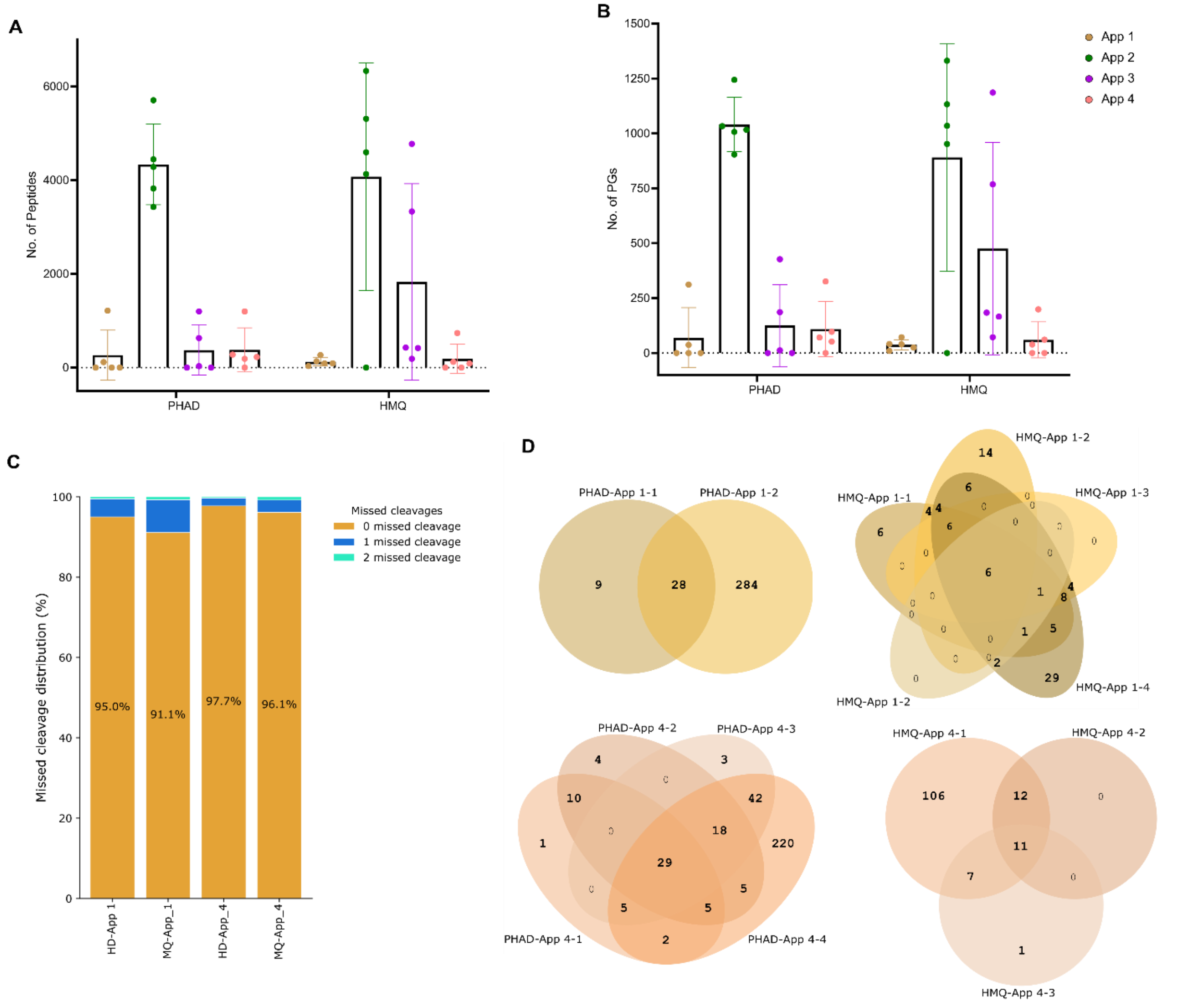

Figure S1: A global comparison of App 1-4**.** The number of identified peptides (A) and PG (B) deparaffinized with PHAD and HMQ. (C) Digestion efficiency from a measure of missed cleavage distribution. (D) Venn diagrams of PG overlap across recovered technical replicates for App_1 and App_4 from PHAD and HMQ deparaffinization.

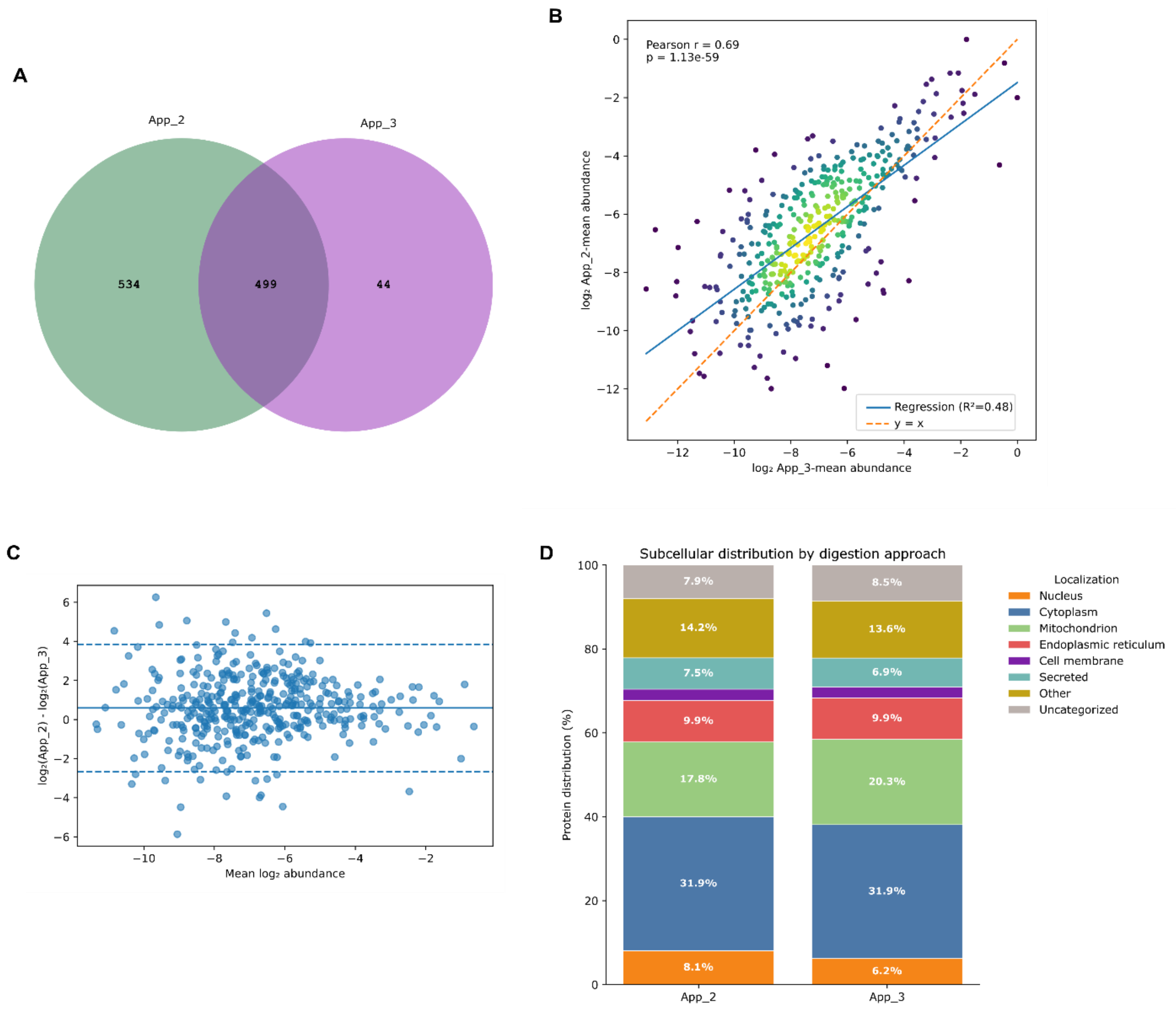

Figure S2: Overlap comparison of App_2 and App_3**.** (A) Venn diagram of unique and shared proteins across App_2 and App_3. (B) A density scatter plot with regression comparing mean abundances of the share 499 PG in App_2 and App_3. (C) A Bland-Altman plot showing abundance differences between the share 499 PG App_2 and App_3. (D) A stacked bar diagram shows a comparison of subcellular distribution of proteins identified by App_2 and App_3.

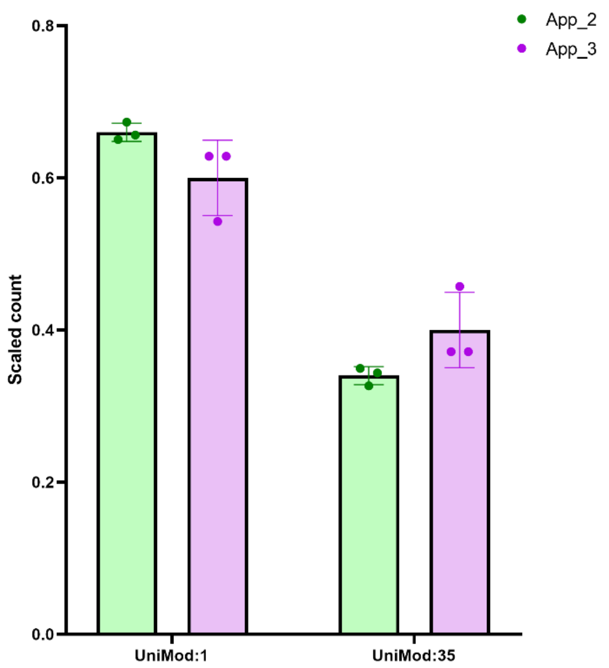

Figure S3: Ratio of posttranslational modified peptides**.** The number of N-terminus acetylation (UniMod:1) and Methionine oxidation (UniMod:35) modified peptides for App_2 and App_3 given as a ratio of the total modified peptides.

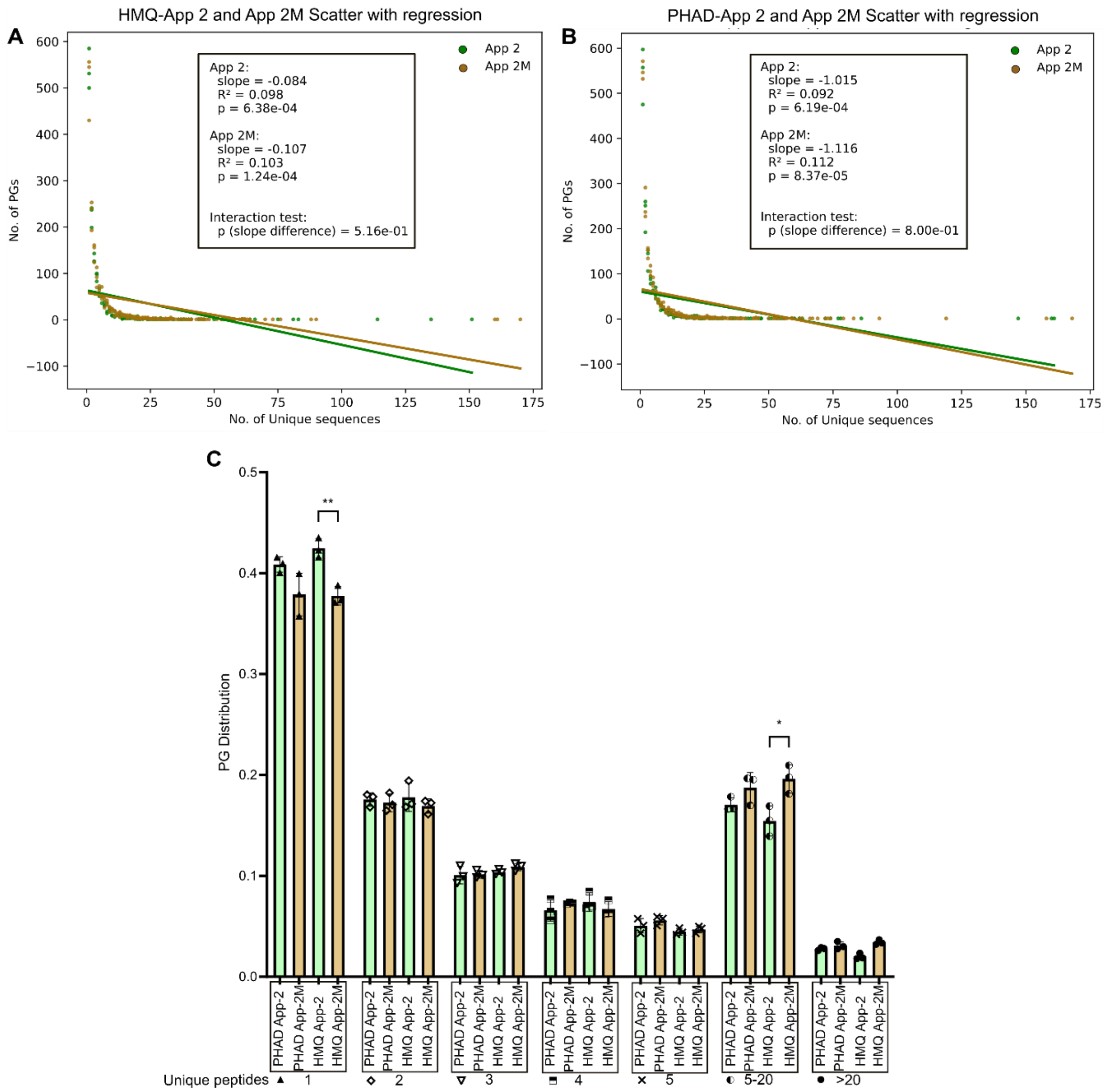

Figure S4: Proportion of proteins identified by unique peptides. Scatter plots showing the relationship between the number of identified proteins and the corresponding number of unique peptides, including fitted regression lines, for HMQ-App_2 and HMQ-App_2M (A), and PHAD -App_2 and PHAD-App_2M (B).

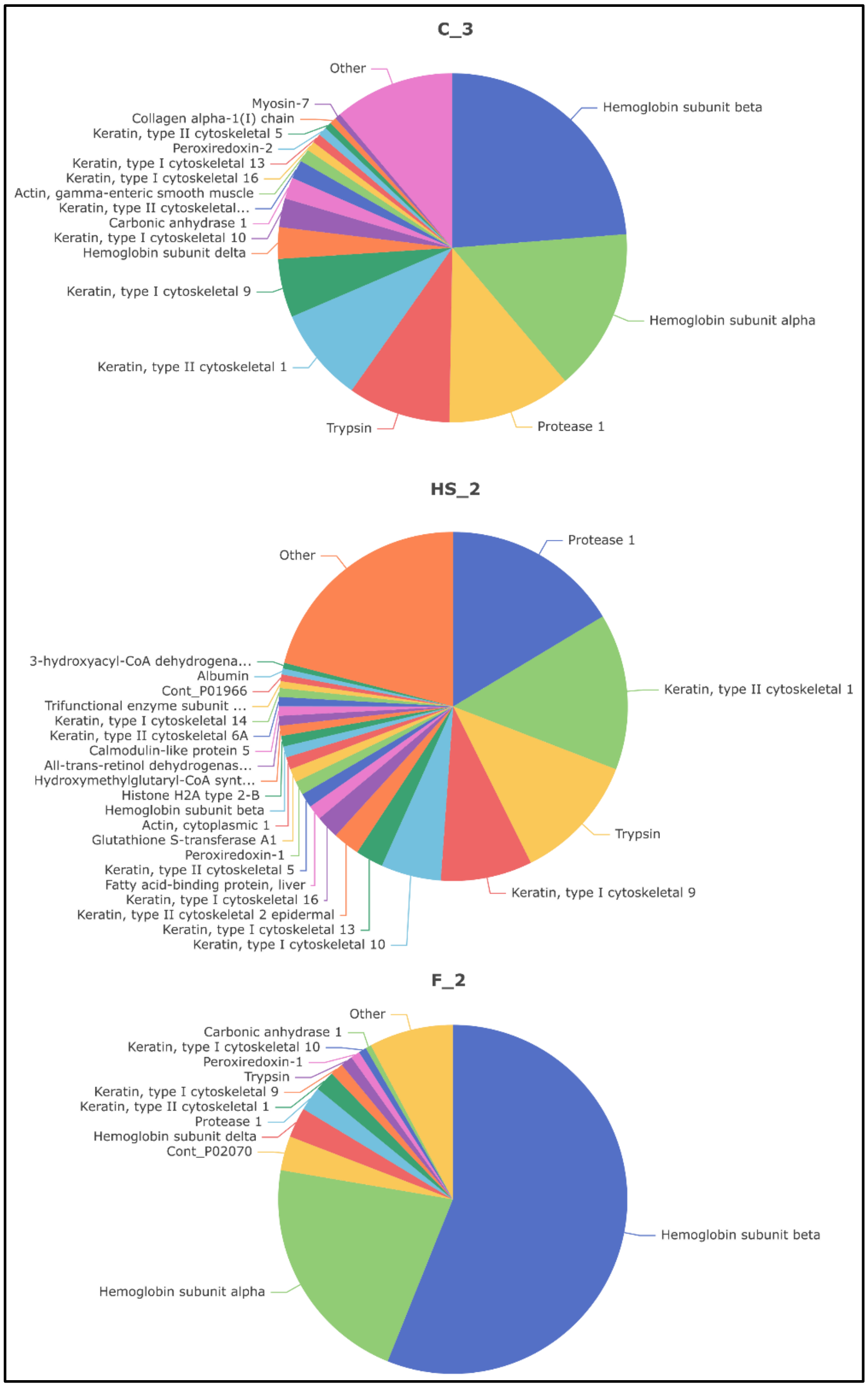

Figure S5: Pie chart showing intensity distribution of protein groups (PGs). Protein group intensities from excluded low-quality samples are displayed as scaled PG.MaxLFQ values and arranged in descending order. Labels C_2, HS_2, and F_2 are replicates of the control, hepatic steatosis, and fibrosis group, respectively.

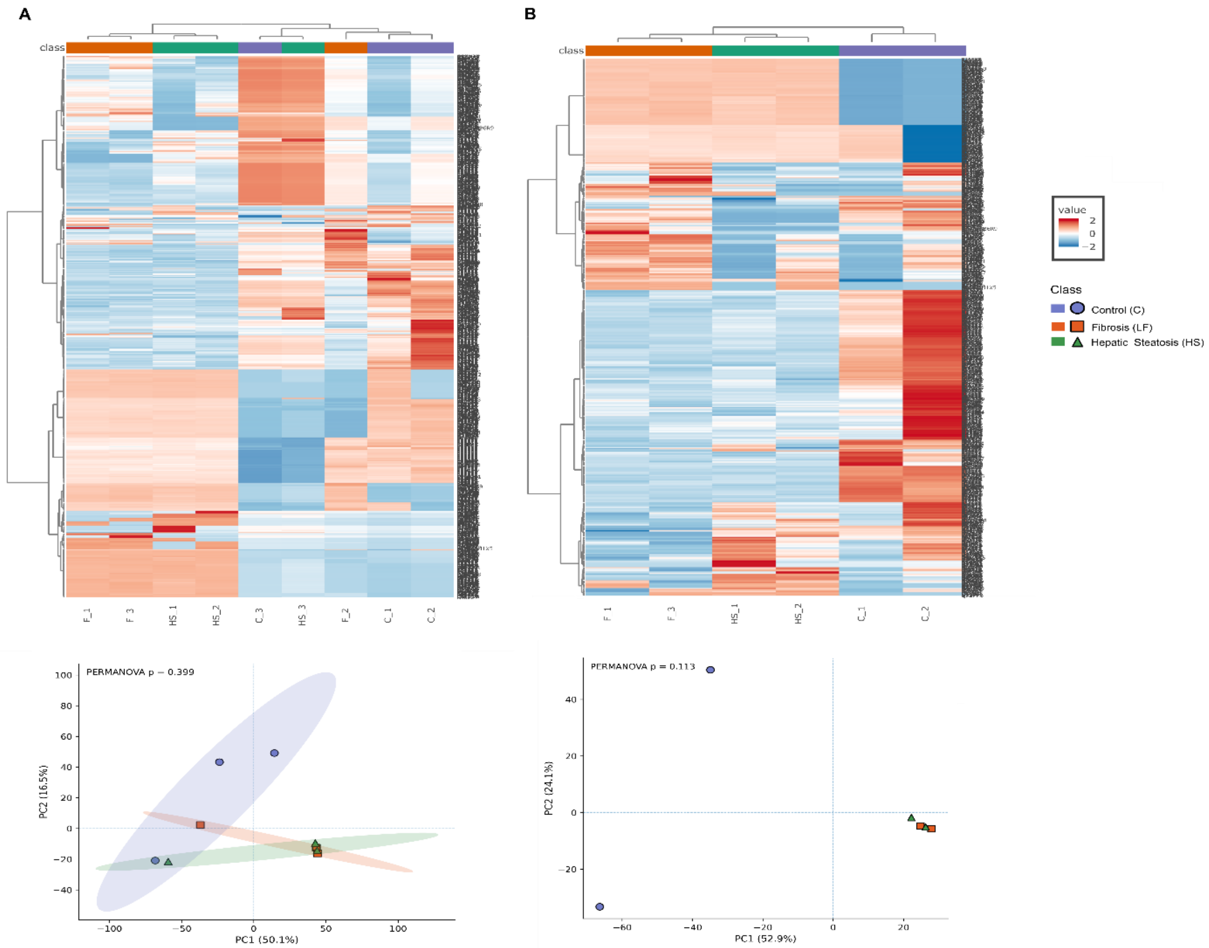

Figure S6: Heatmap and principal component analysis (PCA) of study samples. Clustered heatmap showing sample grouping across control (C_1, C_2), hepatic steatosis (HS_1, HS_2), and fibrosis (F_1, F_3) groups before (A) and after exclusion (B) of low-quality samples, with the corresponding PCA plots presented below.

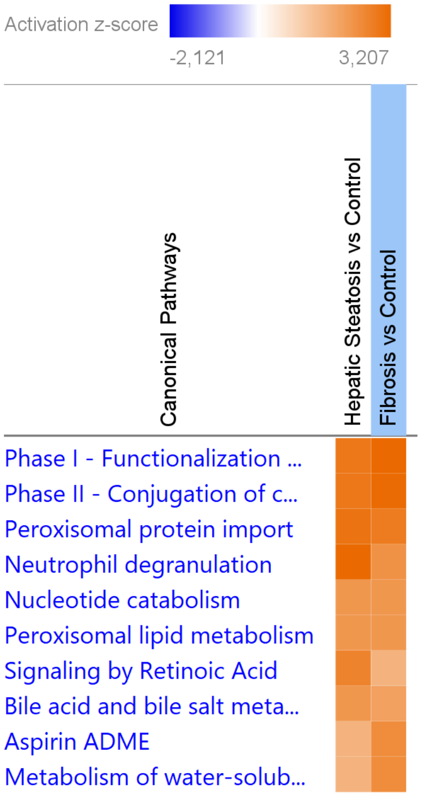

Figure S7: Heatmap of the top 10 canonical pathways derived from differentially abundant proteins (DAPs) in hepatic steatosis and fibrosis. Canonical pathways identified by Ingenuity Pathway Analysis (IPA) are displayed for hepatic steatosis and fibrosis relative to controls. Pathway activity is represented by activation z-scores, with orange indicating predicted activation and blue indicating predicted inhibition; color intensity reflects the magnitude of the z-score.
